# Impact of Preoperative Ureteral Stenting in Retrograde Intrarenal Surgery for Urolithiasis

**DOI:** 10.3390/medicina59040744

**Published:** 2023-04-10

**Authors:** Jae Yong Jeong, Kang Su Cho, Dae Young Jun, Young Joon Moon, Dong Hyuk Kang, Hae Do Jung, Joo Yong Lee

**Affiliations:** 1 Department of Urology, National Health Insurance Service Ilsan Hospital, Goyang 10444, Republic of Korea; urojjy@nhimc.or.kr; 2 Department of Urology, Prostate Cancer Center, Gangnam Severance Hospital, Urological Science Institute, Yonsei University College of Medicine, Seoul 06273, Republic of Korea; kscho99@yuhs.ac; 3 Department of Urology, Severance Hospital, Urological Science Institute, Yonsei University College of Medicine, Seoul 03722, Republic of Korea; dyjun881101@yuhs.ac; 4 Department of Urology, Kyungpook National University Hospital, Kyungpook National University School of Medicine, Daegu 41944, Republic of Korea; jjunny72@knuh.kr; 5 Department of Urology, Inha University College of Medicine, Incheon 22332, Republic of Korea; dhkang@inha.ac.kr; 6 Department of Urology, Inje University Ilsan Paik Hospital, Inje University College of Medicine, Goyang 10380, Republic of Korea; haedojung@paik.ac.kr; 7 Center of Evidence Based Medicine, Institute of Convergence Science, Yonsei University, Seoul 03722, Republic of Korea

**Keywords:** urolithiasis, stents, ureteroscopy

## Abstract

*Background and Objectives*: Ureteral stent insertion passively dilates the ureter. Therefore, it is sometimes used preoperatively before flexible ureterorenoscopy to make the ureter more accessible and facilitate urolithiasis passage, especially when ureteroscopic access has failed or when the ureter is expected to be tight. However, it may cause stent-related discomfort and complications. This study aimed to assess the effect of ureteral stenting prior to retrograde intrarenal surgery (RIRS). *Materials and Methods*: Data from patients who underwent unilateral RIRS for renal stone with the use of a ureteral access sheath from January 2016 to May 2019 were retrospectively analyzed. Patient characteristics, including age, sex, BMI, presence of hydronephrosis, and treated side, were recorded. Stone characteristics in terms of maximal stone length, modified Seoul National University Renal Stone Complexity score, and stone composition were evaluated. Surgical outcomes, including operative time, complication rate, and stone-free rate, were compared between two groups divided by whether preoperative stenting was performed. *Results*: Of the 260 patients enrolled in this study, 106 patients had no preoperative stenting (stentless group), and 154 patients had stenting (stenting group). Patient characteristics except for the presence of hydronephrosis and stone composition were not statistically different between the two groups. In surgical outcomes, the stone-free rate was not statistically different between the two groups (*p* = 0.901); however, the operation time for the stenting group was longer than that of the stentless group (44.8 ± 24.2 vs. 36.1 ± 17.6 min; *p* = 0.001). There were no differences in the complication rate between the two groups (*p* = 0.523). *Conclusions*: Among surgical outcomes for RIRS with a ureteral access sheath, preoperative ureteral stenting does not provide a significant advantage over non-stenting with respect to the stone-free rate and complication rate.

## 1. Introduction

The ureteral stent is an irreplaceable tool for urologists. First described by Herdman in 1949, and later developed in the current ‘double-J’ shape by Thomas Hepperlen and Roy Finney in the 1970s, ureteral stents are commonly used to relieve obstruction of the ureter, prevent complications following upper urinary tract procedures, and provide a scaffold for healing of the ureter. Although a ureteral stent placement serves as the most minimally invasive method for draining urine from the kidney to the bladder, it has some drawbacks, including infection, pain, encrustation, dislodgement, hematuria, and irritative voiding symptoms such as frequency and urgency [1,2].

Since Marshall designed the first flexible ureterorenoscope in 1964, there have been ongoing technological improvements. Technological advances focused on reducing the diameter of the scope while increasing the deflection angle, and it was mainly used for diagnostic purposes. However, during the 1990s, a deflection system with a larger working channel of 3.6 Fr was introduced, and advancements in laser technology using holmium: YAG as a flexible lithotripter became widely employed for retrograde intrarenal surgery (RIRS) in the treatment of upper urinary tract stones during the late 1990s. With its improved stone-free rate (SFR) and low complication rate, treatment indications for RIRS have significantly expanded, and it is now recommended as the first- or second-line treatment for all categories of kidney stones, including stones larger than 20 mm, although multisession treatment may be required according to current treatment guidelines [3,4,5].

RIRS requires repetitive scope insertion to fragment and extract urinary stones; thus, to aid access to the proximal ureter and renal pelvis and to lower intrarenal pressure, a ureteral access sheath (UAS) is widely used during RIRS. However, sometimes ureteral access is not possible—previous studies have reported 8.8% to 20% failure rates for UAS insertion [6]. For these failed cases, or when a tight ureter is expected, double-J ureteral stent insertion is recommended [7], because ureteral stenting induces passive dilation of the ureter, which facilitates passage of the UAS and ureterorenoscope [8]. Furthermore, ureteral stent insertion is sometimes needed before RIRS to decompress obstruction of the collecting system or to relieve renal colic [9,10].

In this regard, some studies have analyzed the relationship between RIRS and preoperative ureteral stenting, reporting that preoperative ureteral stent placement improves the SFR [11,12,13]. However, other studies have reported no statistical differences in the SFR between preoperative stenting groups and stentless groups [14,15,16]. In addition, the impacts of preoperative stenting on operative time and complication rates were not clear. Thus, we analyzed patients at our single tertiary medical center to evaluate the effects and efficacy of preoperative stenting before RIRS.

## 2. Materials and Methods

### 2.1. Study Population

We retrospectively reviewed patients who had RIRS for ureteral or renal stones from January 2016 to May 2019. The decision to treat the stone surgically was made according to EAU guidelines, which include symptomatic ureteral stones, growing renal stones, obstruction caused by stones, and infection [17]. The decision to perform preoperative ureteral stenting was made by the surgeon (J.Y.L) who performed the actual surgery.

**Figure 1:**
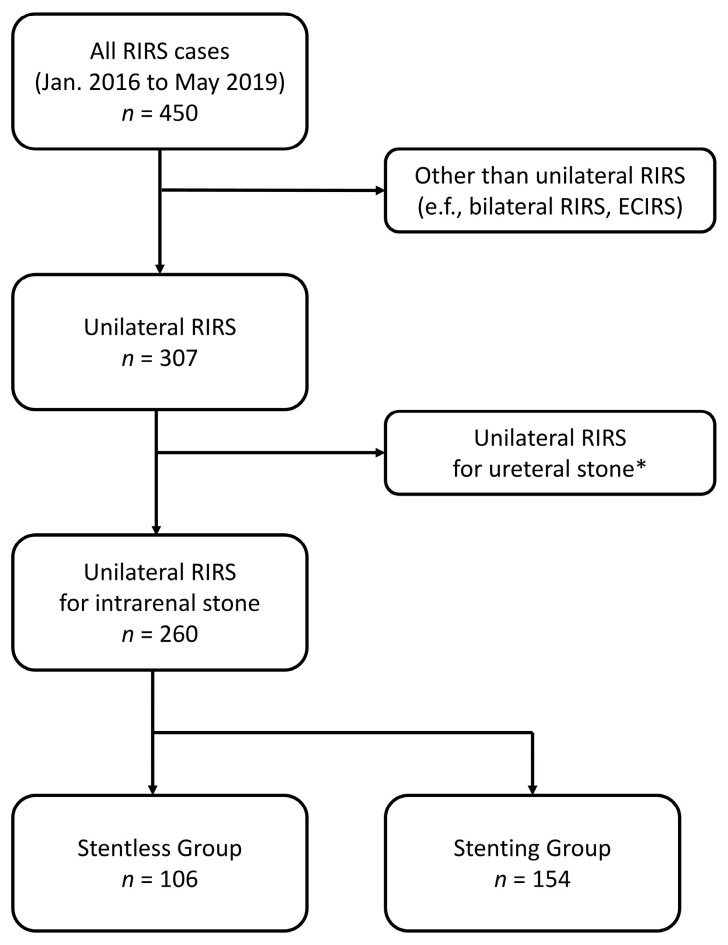
Flowchart of our retrospective study design. RIRS = retrograde intrarenal surgery, ECIRS = endoscopic combined intrarenal surgery. * Confirmed by surgical record and routinely performed preoperative CT imaging.

**Figure 2:**
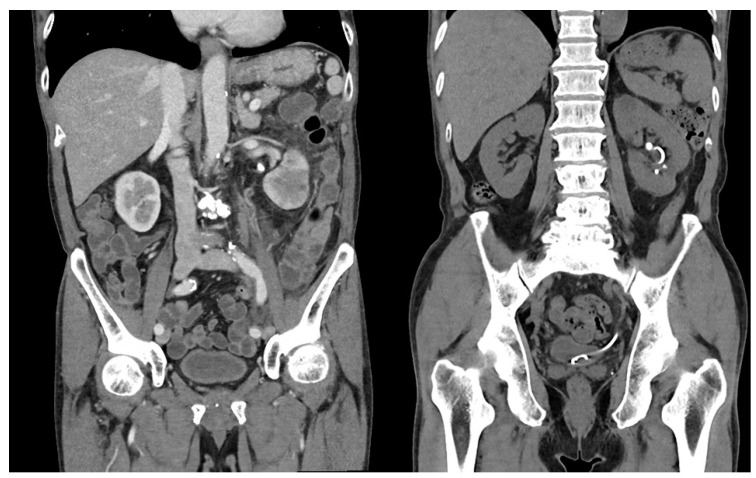
Initial and preoperative CT images of a patient presenting with a proximal ureteral stone with infection. In the right image, the stone migrated to the upper calyx with the inserted ureteral stent. **This patient had an mS-ReSC score of 5 (one stone in the renal pelvis and two stones in the inferior minor calyces**).

Firstly, we collected cases in which RIRS was performed for a proximal ureter or renal stone. For an accurate comparison of surgical outcomes of RIRS, we excluded cases that were not unilateral RIRS (e.g., bilateral RIRS, endoscopic combined intrarenal surgery) and included only cases carried out for renal stones by checking low-dose non-contrast stone CT imaging routinely performed within 3 days of surgery and the surgical records. However, stones that were ureteral stones before stent placement but were found to be located inside the kidney after stent placement were included (Figure 1 and Figure 2).

Patient characteristics, including age, sex, body mass index (BMI), presence of hydronephrosis, and treated side, were obtained. Hydronephrosis was identified using preoperative computed tomography (CT) diagnostic imaging. Maximal stone length (MSL) and modified Seoul National University Renal Stone Complexity (mS-ReSC) score were set as stone characteristics. MSL was measured via CT imaging in bone windows/level settings and the mS-ReSC score was assigned according to the number of sites involved in the renal pelvis (#1), superior and inferior major calyceal groups (#2–3), and anterior and posterior minor calyceal groups of the superior (#4–5), middle (#6–7), and inferior calyx (#8–9). If the stone was in the inferior sites (#3, #8–9), one additional point per site was added to the original score (see the bold in Figure 2) [18]. Lastly, the stone composition data were recorded and stratified into six groups according to the Mayo Clinic classification system [19,20]. All stone fragments obtained at this medical center were sent to GC Laboratories, Yongin, Korea, for quantitative analysis of stone composition through FTIR spectroscopy.

This study protocol was reviewed and approved by the Institutional Review Board of Severance Hospital, Yonsei University Health System, Seoul, Korea (Approval No. 4-2022-1568; approval date: 1 February 2023). However, the requirement for written informed consent of subjects was waived due to the anonymization of patient data and the retrospective study design.

### 2.2. Surgical Technique for Retrograde Intrarenal Surgery

In cases that used preoperative stenting, cystoscopic ureteral stent insertion (6-Fr Double-J, Polaris™ Ultra or Polaris™ Loop; Boston Scientific, Marlborough, MA, USA) was performed under local anesthesia by various urological department residents. As usual, the ureteral stent was inserted using a cystoscope and guidewire in the lithotomy position.

RIRS was performed under general anesthesia as follows. The patient was placed in the lithotomy position, and a 0.035′′ flexible hydrophilic-coated guidewire (Roadrunner^®^ Wire Guide; Cook Medical, Bloomington, IN, USA) was introduced as a safety guidewire into the renal pelvis in a retrograde fashion. If preoperative ureteral stenting was performed, a hydrophilic guidewire was inserted through the previously inserted ureteral stent. A dual-lumen ureteral catheter (Dual Lumen, Boston Scientific) was advanced over the safety guidewire; retrograde pyelography was performed; and then a stiff guidewire (Amplatz Super Stiff Guidewire, Boston Scientific) was placed next to the safety guidewire. An 11/13-Fr (Uropass; Olympus Corp., Tokyo, Japan or Navigator, Boston Scientific) was advanced into the proximal ureter over the stiff guidewire. A flexible uretero-reno videoscope was inserted through the UAS. Four kinds of scopes were used, as follows: FLEX-XC digital flexible video ureterorenoscope (KARL STORZ Endoskope, Tuttlingen, Germany), URF-V and URF-V2 flexible video ureteroscopes (Olympus Corp.), and LithoVue™ single-use digital flexible ureteroscope (Boston Scientific). Lithotripsy was performed with a holmium: YAG laser lithotripter (VersaPulse™ PowerSuite™ 100W; Lumenis, Tel Aviv, Israel) using 200-micron laser fibers. Depending on the size and hardness of the stone, fragmentation and dusting methods were utilized appropriately. Large, fragmented stones were extracted with a 1.9-Fr Zero Tip™ Nitinol Stone Basket (Boston Scientific). Stone dust particles were not removed, as they were expected to drain naturally. A 6-Fr double-J ureteral stent was routinely placed after the RIRS procedure and maintained for 1 to 2 weeks in all patients. In all cases, surgical procedures were performed by the same experienced single surgeon (J.Y.L.). After removal of the postoperative stent on an outpatient basis, follow-up non-contrast CT was performed at 1 to 3 months, and the presence or absence of residual stones confirmed the stone-free status.

### 2.3. Statistical Analyses

The patients were divided into two groups. One group did not have a preoperative ureteral stent placed prior to RIRS (stentless group), whereas the other group had a double-J ureteral stent placed prior to RIRS (stenting group). Patient demographics, stone characteristics, and surgical outcomes were compared between the two groups. Surgical outcomes included operative time, postoperative complications categorized according to the Clavien–Dindo classification system [21], and SFR. In this study, stone-free status was defined as either having no identifiable stones or the remaining stone fragments were <4 mm on the follow-up postoperative CT scan.

Data are presented as the mean ± standard deviation unless otherwise indicated. Dichotomous and categorical variables are presented as actual numbers and as percentages of the total population. Student’s two-sample *t*-test was used for statistical comparisons of continuous demographic variables. The Shapiro–Wilk test was performed to check the distribution of continuous variables, and the Man n–Whitney test was performed if the normal distribution was not met. Pearson’s chi-squared test with Yates’s correction for continuity was used to compare categorical variables. Univariate and multivariate logistic regression with a binomial method were performed to analyze factors affecting the SFR. All computations were performed using R version 4.2.1 (R Foundation for Statistical Computing, Vienna, Austria; http://www.r-project.org; accessed on 26 June 2022).

## 3. Results

Included in this study were 260 patients who underwent unilateral RIRS for intrarenal stone. The patients were divided into two groups: 106 patients who had no stent insertion before RIRS were classified as the stentless group, and 154 patients who had preoperative ureteral stent insertion were classified as the stenting group.

In the demographics of patient and stone characteristics, there were no statistically significant differences between the two groups other than the presence of hydronephrosis and the stone composition group (*p* = 0.015 and *p* = 0.025, respectively). The rate of hydronephrosis present on preoperative CT scans was higher for the stenting group than for the stentless group (62.3% vs. 46.2%). Calcium oxalate stones were the most commonly occurring stone type in both groups; however, uric acid stones were more common in the stenting group than in the stentless group (28.6% vs. 15.1%) (Table 1).

Regarding surgical outcomes, operation time was longer for the stenting group than for the stentless group (44.8 ± 24.2 vs. 36.1 ± 17.6, *p* =0.001). However, the SFR and postoperative complication showed no statistical difference between the two groups (*p* = 0.901 and *p* = 0.523, respectively) (Table 2). In the complication category, two patients in each group experienced postoperative sepsis requiring inotropes, which improved with continued IV antibiotics. Two patients in the stenting group required postoperative blood transfusion. One Clavien–Dindo grade 3a patient who had a postoperative cystoscopic ureteral stent exchange under local anesthesia due to severe pain was in the stentless group.

Univariate logistic regression models revealed a shorter MSL and lower mS-ReSC score as statistically significant factors affecting the SFR (*p* < 0.001, both groups). A multivariate analysis corroborated a shorter MSL (*p* = 0.006) and lower mS-ReSC score (*p* < 0.001) as independent significant factors for the SFR (Table 3).

## 4. Discussion

A ureteral stent is mainly used to help maintain urine drainage in cases of obstructive uropathy. This medical device is composed of a flexible tube with small side openings that is inserted into the ureter [22]. Ureteral stent placement also induces passive ureteral dilation, which is reversible. The exact mechanism has not been elucidated, but it may be caused by physiologic relaxation or direct cytotoxic effects. Dilation of the ureter appears to occur when foreign material (such as a stent) is present in the ureter, although dilation may be related to alterations in the renal pelvis or ureteral peristalsis induced by the stent, thus slowing down urine transport. Ureteral dilation is also associated with an inflammatory response of the ureteral wall [23,24]. Meanwhile, as RIRS has been proposed as one of the first line treatment options for stones smaller than 20 mm, various studies have been published on the relationship between RIRS and ureteral stents.

Previous studies have suggested that preoperative ureteral stenting may be beneficial for RIRS surgery by inducing passive dilatation [15,16]. However, randomized controlled trials on this topic are lacking and current EAU urolithiasis guidelines do not recommend routine preoperative ureteral stenting. Therefore, we analyzed the data from our institution to compare the effect of preoperative stenting on RIRS and found that there was no statistical difference in SFR and postoperative complication rate.

Several previous studies favoring preoperative stenting did not distinguish differences such as the use of flexible scopes or the use of UAS [11,12,13]. To exclude these confounders, we restricted our study to unilateral RIRS which used UAS during procedure. Under similar conditions, the study by Chu et al. showed a higher SFR in the preoperative stenting group. One of the reasons cited for this outcome included the ability to insert a larger-size UAS by ureteral passive dilation, which would have allowed for more efficient irrigation during ureteroscopy, leading to better visualization and effective fragment washout. They used a larger-size UAS (14/16 Fr) with the preoperative stenting group compared with the non-stented group (12/14 Fr). Like Chu et al., Rubensein et al. also showed a higher SFR in the preoperative stenting group, where the proportion of larger-size (14/16 Fr) UAS used was higher in the preoperative stenting group. In contrast, all UAS were the same 11/13 Fr in our data. Given our institution’s routine practices and to reduce confounding factors, we limited our analysis to RIRS using the same 11/13 Fr UAS, which may have offset the effect of passive ureteral dilation on SFR. In the study by Lumma et al. that did not use UAS, the SFR was higher in the preoperative stenting group [12].

Although Law et al. reported in their meta-analysis that preoperative stenting improves SFR in the ureterorenoscopic treatment of renal stones [25], the comparison between the two groups in terms of stone characteristics was not clear enough, which may have affected the results. For example, Assimos et al. conducted a good global observational study, but, in their study, cases with both ureteral and renal stones were classified as renal cases, and it was not clear what kind of scope was used for cases with both renal and ureteral stones [26]. Given this, we limited our study design to cases with renal stones only, and, for ureteral stones, we included only cases in which the stone moved inside the kidney during the stent insertion process. After excluding ureteral stones, we introduced the mS-ReSC score to assess the impact of stone location. In terms of stone length, Chu et al. and Netsch et al. performed a subgroup analysis [13,15]. However, in this study, we did not perform a subgroup analysis because the stone size in both groups averaged 11 mm (11.2 ± 5.4 vs. 11.9 ± 5.4, *p* = 0.353), which was adequate for the surgical indication of RIRS.

We also collected only cases performed by a single, sufficiently skilled surgeon to exclude the effect of differences between operators. In our medical center, four kinds of flexible uretero-reno videoscopes were used and three of which were re-usable (FLEX-XC digital flexible video and URF-V and URF-V2 flexible video ureteroscopes), whereas one was disposable for single use (LithoVue™). The type of scope was not distinguished when collecting data according to a systematic review that found no significant differences in surgical outcomes including SFR, complication rate, operation time, and hospital stay between the use of reusable and disposable flexible ureteroscopes for stone surgeries [27].

Logistic regression modeling revealed that preoperative stenting was not the independent predictor of SFR in our study, which was consistent with the previous studies [28,29]. This might suggest that if a flexible ureteroscope is able to enter the renal pelvis, stone characteristics including stone length and location of stone are more influential than performance benefits such as easier stone removal with basket device and stone particle passage. Ito et al. reported that this might be due to the relatively large stone volume and number of cases in preoperative stenting due to the nature of tertiary medical centers. However, in our study, there was no significant difference in terms of stone size between the stentless and the stenting groups (11.2 ± 5.4 vs. 11.9 ± 5.4, *p* = 0.353), and the stenting group also had a higher proportion than in their study (59.2% vs. 46.1%).

The finding of no difference in postoperative complication rates with and without stenting was consistent with that of a meta-analysis by Law et al. [25]. Upon review of the surgical records and subsequent medical records, no iatrogenic complications such as ureteral perforation or avulsion were found in both groups, although we presented only postoperative complication according to CD classification in the results.

The present study found that operative time was longer in the stenting group. The same results were found in some studies and Lumma et al. suggested that this could be attributed to stent extraction prior to RIRS [12,26]. In contrast, Zhang et al. reported no difference in operative time and cited a higher proportion of ureteral stone cases as a reason [28]. Chu et al. also showed that operative time was significantly shorter in the stenting group (93.3 ± 39.9 vs. 123.6 ± 59.8, *p* = 0.008) [15]. They attributed this to the availability of a larger UAS and a less narrow ureteropelvic junction and a straighter ureter in the presented group, which may have allowed for easier access and stone retrieval. In our opinion, this difference might be due to a selection bias caused by the retrospective study design. Patients who needed stent insertion before surgery might have been more likely to have a complicated stone. In addition, preoperative CT images revealed more cases of hydronephrosis in the stenting group than in the stentless group, which may have caused differences in scope movement during the RIRS procedure affecting operative time. Furthermore, there was a difference in stone composition between the two groups. The proportion of calcium oxalate stones, which are the most common, was higher in the stentless group (49.1% vs. 36.4%), but the proportion of struvite stones and uric acid stones, which are relatively large, was higher in the stenting group (29.2% vs. 31.8% and 15.1% vs. 28.6%). One thing to consider, however, is that, in the Chu et al. study, the time spent on preoperative stenting was included in the operative time. Under the same conditions, the results of this study might be different.

The data for this study was limited to January 2016 through May 2019. As we have gained sufficient experience with safe access sheath insertion and digital flexible ureteroscopy, we have seen fewer cases of failed ureteral entry or ureteral injury on the first attempt. Therefore, we have gradually reduced preoperative ureteral stenting in our institution, which can cause unnecessary hematuria, UTIs, and urolithiasis in patients scheduled for surgery [30,31]. In addition, a systematic review published by EULIS in 2020 recommended limiting preoperative stent dwell time to prevent UTI or urosepsis in post-operative ureteroscopy for stone disease [32], which further reduced preoperative stenting.

Another limitation of this retrospective study is that ureteral stent insertion was not randomized. Assimos et al., in their study, presented a predictive model for preoperative ureteral stent placement. In general, clinicians preferred to place preoperative ureteral stents in cases with high comorbidities such as high ASA score, solitary kidney, anticoagulant use, and Crohn’s disease [26]. We performed stenting if the ureteral stone was causing symptoms, such as renal colic, obstructive uropathy, or infection, in accordance with present guidelines [17]. In this tertiary medical center, these were mostly patients who were referred to urology on an outpatient basis, in the emergency room, or as a consultation from another department. They were subsequently scheduled for surgery under general anesthesia to remove the stone after stone-related symptoms resolved. In the renal stone group, ureteral stenting was considered first in younger patients with a relatively high likelihood of failed ureteroscopy [33], and was otherwise considered upon patients’ will; it was only performed in patients who were informed of the benefits and complications of ureteral stenting and readily agreed to it.

A well-designed randomized controlled trial is needed to address these shortcomings and validate the results. However, despite these limitations, we aimed to eliminate confounding factors from previous studies through more detailed study design, which might help strengthen existing guidelines to prevent unnecessary discomfort and additional costs from preoperative ureteral stent insertion.

## 5. Conclusions

Among patients who underwent RIRS with a UAS, those who had preoperative ureteral stenting did not show a significant difference in SFR and complication rate compared with those who did not. Therefore, except when necessary to resolve obstructive uropathy or renal colic, routine preoperative stenting is not needed to improve surgical outcomes of RIRS.

## Figures and Tables

**Table 1:** Summary of patients and stone characteristics by preoperative stenting status.

	Stentless Group (*n* = 106)	Stenting Group (*n* = 154)	*p*-Value
Age	58.5 ± 14.0	57.6 ± 13.9	0.608 ^a^
Sex			0.415 ^b^
-Male	55 (51.9%)	89 (57.8%)	
-Female	51 (48.1%)	65 (42.2%)	
BMI	24.9 ± 3.8	25.2 ± 5.6	0.559 ^a^
Laterality			0.754 ^b^
-Left	58 (54.7%)	80 (51.9%)	
-Right	48 (45.3%)	74 (48.1%)	
Hydronephrosis	49 (46.2%)	96 (62.3%)	0.015 ^b^
MSL (mm)	11.2 ± 5.4	11.9 ± 5.4	0.353 ^a^
mS.ReSC score	2.2 ± 1.5	2.5 ± 1.6	0.115 ^a^
Stone composition group			0.25 ^b^
-Struvite	31 (29.2%)	49 (31.8%)	
-Cystine	0 (0.0%)	0 (0.0%)	
-Uric acid	16 (15.1%)	44 (28.6%)	
-Brushite	1 (0.9%)	0 (0.0%)	
-Calcium oxalate	52 (49.1%)	56 (36.4%)	
-Carbonate apatite	6 (5.7%)	3 (1.9%)	
-Others	0 (0.0%)	2 (1.3%) *	

BMI = body mass index, MSL = maximal stone length, mS.ReSC = modified Seoul National University Renal Stone Complexity; * One ammonium urate and one protein were identified; ^a^ Based on Student’s two-sample *t*-tests. The Shapiro–Wilk test was performed to check the distribution of continuous variables, and the Mann-Whitney test was performed if the normal distribution was not met. ^b^ Based on Pearson’s chi-squared tests with Yates’s correction for continuity.

**Table 2:** Differences in surgical outcomes with and without preoperative stenting.

	Stentless Group (*n* = 106)	Stenting Group (*n* = 154)	*p*-Value
Operative time	36.1 ± 17.6	44.8 ± 24.2	0.001 ^a^
Complication			0.523 ^b^
-CD grade 1	16 (15.1%)	18 (11.7%)	
-CD grade 2	2 (1.9%)	4 (2.6%)	
-CD grade 3a	1 (0.9%)	0 (0.0%)	
-CD grade 3b	0 (0.0%)	0 (0.0%)	
Stone-free rate *	97 (91.5%)	189 (90.3%)	0.901 ^b^

CD = Clavien–Dindo; * Stone-free rate means no residual stone or stone fragments of <4 mm in size on follow-up computed tomography image. ^a^ Based on Student’s two-sample *t*-test. The Shapiro-Wilk test was performed to check the distribution of continuous variables, and the Mann-Whitney test was performed if the normal distribution was not met. ^b^ Based on Pearson’s chi-squared tests with Yates’s correction for continuity.

**Table 3:** Logistic regression models for predicting stone-free rate according to test parameters.

	Odds Ratio	95% CI	*p*-Value
Univariate			
Hydronephrosis	1.031	0.570–0.853	0.917
Preoperative stenting	0.942	0.514–1.701	0.845
MSL	0.884	0.836–0.932	<0.001
mS-ReSC	0.635	0.527–0.759	<0.001
Composition group *			
Struvite	0.726	0.395–1.360	0.31
Uric acid	1.194	0.600–2.525	0.625
Calcium oxalate	1.008	0.559–1.838	0.978
**Multivariate**			
MSL	0.920	0.865–0.976	0.006
mS-ReSC	0.707	0.578–0.860	<0.001

MSL = maximal stone length (mm), mS-ReSC = modified Seoul National University Renal Stone Complexity. * Other groups were excluded due to the small number of samples.

## Data Availability

Data are available upon request to the corresponding author.
